# Should Assessments of Decision-Making Capacity Be Risk-Sensitive? A Systematic Review

**DOI:** 10.3389/fpsyg.2022.897144

**Published:** 2022-06-29

**Authors:** Noah Clark Berens, Scott Y. H. Kim

**Affiliations:** Department of Bioethics, Clinical Center, National Institutes of Health, Bethesda, MD, United States

**Keywords:** bioethics, decision-making capacity for treatment, review – systematic, capacity, mental competency

## Abstract

**Background:**

The concept of decision-making capacity (DMC) or competence remains controversial, despite widespread use. Risk-sensitive DMC assessment (RS-DMC)—the idea that the higher the risk involved in a decision, the greater the decisional abilities required for DMC—has been particularly controversial. We conducted a systematic, descriptive review of the arguments for and against RS-DMC to clarify the debate.

**Methods:**

We searched PubMed/MEDLINE (National Library of Medicine), PsycInfo (American Psychological Association) and Philpapers, updating our search to February 15th, 2022. We targeted peer-reviewed publications in English that argue for or against RS-DMC. Two reviewers independently screened the publications and extracted data from each eligible manuscript.

**Results:**

Of 41 eligible publications, 22 supported a risk-sensitive threshold in DMC assessment. Most arguments for RS-DMC rely on its intuitive appeal and practical merits. The arguments against RS-DMC primarily express concerns about paternalism and the seeming asymmetry between consent and refusal; critics of RS-DMC support epistemic, rather than substantive (i.e., variable threshold), risk-sensitivity; counterarguments responding to criticisms of RS-DMC address charges of paternalism and exhibit a notable variety of responses to the issue of asymmetry. Authors used a variety of frameworks regarding the definition of DMC, its elements, and its relation to decisional authority, and these frameworks were significantly associated with positions on RS-DMC. A limitation of our review is that the coding relies on judgment and interpretation.

**Conclusion:**

The review suggests that some of the debate about RS-DMC stems from differences in underlying frameworks. Most defenses of RS-DMC rely on its intuitive appeal, while most criticisms reflect concerns about paternalism or the asymmetry between consent and refusal. Defenses of RS-DMC respond to the asymmetry problem in a variety of ways. Further research is needed on the implications of underlying frameworks, the asymmetry problem, and the distinction between epistemic and substantive models of RS-DMC.

## Introduction

In most jurisdictions, decision-making capacity (DMC) is used to classify patients into two groups: those whose medical decisions should be made by the patient herself and those whose decisions need to be made by another party. Thus, faulty assessment of DMC can result in either failure to protect a vulnerable incapacitated patient from harm or violation of a capacitated patient's autonomy. Despite its importance, the concept of DMC remains controversial. Issues such as how emotions affect DMC (Charland, 1998), whether the ability to value is relevant to DMC (Kim, 2010), how authenticity should play a role (den Hartogh, 2016; Ahlin Marceta, 2020), and the role of voluntariness have all inspired debate (Charland, 2002). A recent narrative review explores the role of emotions and values, and highlights the complexity and lack of consensus (Hermann et al., 2016).

One particularly controversial point of debate is whether DMC assessment should be risk-sensitive. Risk is a broad term that refers to the seriousness or momentousness of a decision. Thus, risk-sensitive assessment of DMC (RS-DMC) refers to the idea that when the stakes of a decision are high, the level of decisional abilities needed for DMC should be higher as well (Drane, 1984; Buchanan and Brock, 1986; Culver and Gert, 1990). As described in an English legal decision, what matters is whether “[the patient] had a capacity which was commensurate with the gravity of the decision which he purported to make. The more serious the decision, the greater the capacity required” [Re T (Adult: refusal of medical treatment), 1992]. For example, a patient deciding whether or not to withdraw life-sustaining treatment may be held to a high threshold, while a patient deciding between two similarly effective antibiotics may be held to a lower one.

Debate over whether RS-DMC is appropriate seems to have begun in the literature in 1984 (Drane, 1984), yet remains unresolved. Despite persistent disagreement in the literature, RS-DMC is widely accepted and used by clinicians (Kim et al., 2006). The concept has been frequently referenced in UK legal decisions (Buchanan, 2004; Parker, 2006). If RS-DMC is ethically problematic, therefore, it has broad implications for both clinical practice and the law. Furthermore, a wide variety of definitions, concepts, and arguments are used in the literature, making the debate particularly complex. This lack of clarity could lead to inconsistency in DMC evaluation in clinical practice. Accordingly, we conducted a systematic review of the arguments for and against RS-DMC. Our aim is descriptive, so we do not aim to resolve the disagreements about RS-DMC, but to clarify the wide variety of issues at hand in order to promote future fruitful debate.

## Methods

### Search Strategy

We used the Preferred Reporting Items for Systematic Reviews and Meta-analyses (PRISMA) checklist (Page et al., 2021). One author (NB) searched the following citation and abstract databases from inception until August 5th, 2021: PubMed/MEDLINE (US National Library of Medicine), Philpapers, and PsycInfo (American Psychological Association). The search strategy used keywords and controlled vocabulary terms (MeSH and Thesaurus of Psychological index Terms) for the topic of interest. The searches were limited to English language and peer-reviewed publications only where possible. The full search strategy (Supplementary Materials) was reviewed and validated by an independent librarian from the National Institutes of Health Library, and the search was updated to February 15th, 2022. Finally, we used the snowball method and our own experience to add publications that were not detected in the three databases. All search results were exported to EndNote X9 and duplicate citations were identified and removed.

### Inclusion and Exclusion Criteria

We included an article if:

1) It argues for or against risk-sensitive assessment of decision-making capacity^1^
2) The publication is peer-reviewed
3) The publication is written in English

We excluded purely descriptive publications that do not make an argument for either position. We did not require that publications focus exclusively or predominantly on the issue of RS-DMC, only that they make an argument for or against RS-DMC. Additionally, because some of the initial discussion about RS-DMC originated in books, we eventually included five books that appear in the debates or were known to us in the literature.

### Study Selection and Data Extraction

To minimize bias, two readers (NB and a research assistant) independently performed the title/abstract screening and the full-text screening following the predefined inclusion and exclusion criteria. Discrepancies were resolved through discussion until agreement was reached, and when needed, a third reader (SK) assisted in this process. We identified and extracted frameworks and reasons as follows. Both authors (NB, SK) read 50% of the eligible manuscripts and independently created coding schemas. After comparison and discussion, a preliminary schema was agreed upon and adjusted as necessary while coding the eligible manuscripts. The resulting coding scheme had two parts: frameworks, which refer to structural or content features of a publication's conception of concepts related to RS-DMC, and specific reasons used in a publication. Both authors identified passages in each publication that constituted a “reason” or “framework.” Reasons were categorized into three groups: (1) Arguments for RS-DMC, (2) Arguments against RS-DMC, and (3) Counterarguments defending RS-DMC^2^. This categorization naturally emerged from the progression of the debate in the literature, as the first publications discussing RS-DMC defended it, and were then critiqued, opening the door for counterarguments. Thus, our categories reflect the progression of the dialogue characteristic of the debate. In addition, we tracked the year of publication, type of journal, and author background.

## Results

**Figure 1:**
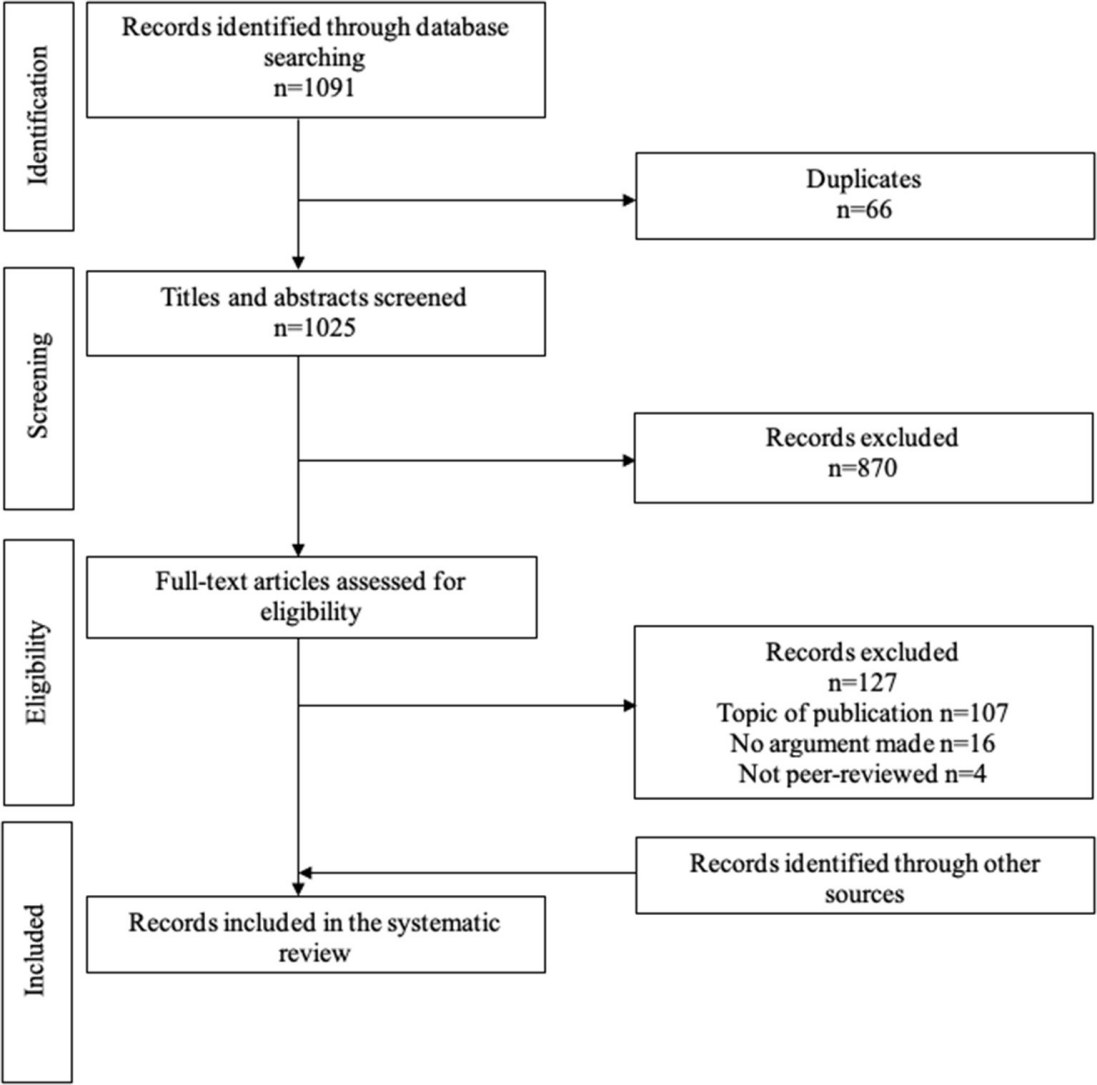
PRISMA flow chart for article selection.

The systematic search yielded 1,058 articles (Figure 1). Of those, 28 were eligible for inclusion. We identified 13 additional eligible publications, for a total of 41 publications (36 articles and 5 books) (Drane, 1984, 1985; Buchanan and Brock, 1986, 1989; Feinberg, 1986; Eastman and Hope, 1988; Kloezen et al., 1988; Culver and Gert, 1990; Brock, 1991; Elliott, 1991; Saks, 1991, 1999; Skene, 1991; Wicclair, 1991a,b, 1999; Winick, 1991; Schopp, 1994; White, 1994; Wilks, 1997, 1999; Grisso and Appelbaum, 1998; Cale, 1999; Maclean, 2000; Berghmans, 2001; Buller, 2001; Checkland, 2001; DeMarco, 2002; Buchanan, 2004; Parker, 2004, 2006; Saks and Jeste, 2006; Howe, 2010; Kim, 2010; Bolt and van Summeren, 2014; Brudney and Siegler, 2015; Manson, 2015; den Hartogh, 2016; Lawlor, 2016; Roberts, 2018; Graber, 2021).

### Publication Characteristics

Dates of publication ranged from 1984 and 2021, though most (32/41) were published between 1984 and 2006. Most articles were published in Bioethics/Philosophy journals (20/36), followed by Clinical journals (6/36) and Law/Policy journals (5/36). Nonclinical (philosophy, bioethics, law, policy), was the most common author background (25/41), followed by mixed background (10/41) and clinical (medicine, psychiatry, etc.) background (6/41).

### Views on RS-DMC

Three main stances emerged: (1) the substantive view, according to which the threshold required for competence itself should vary with risk, (2) the epistemic view, according to which the amount of evidence or certainty required for a finding of competence should vary with risk, and (3) neither (Table 1). A slight majority (22/41) supported the substantive view of RS-DMC, while fewer supported the epistemic view only (12/41). Most publications in clinical journals (5/6) or books (4/5) supported the substantive view. Notably, most articles or books published before 1990 held the substantive view (6/8 publications). All epistemic view articles were published in 1990 or later.

**Table 1:** Views on RS-DMC.

**View**	**Definition**	***N***	**References**
**Views on RS-DMC**			
View on RS-DMC	*Substantive* The threshold required for competence itself should vary with risk.	22	Drane, 1984, 1985; Buchanan and Brock, 1986, 1989; Feinberg, 1986; Eastman and Hope, 1988; Brock, 1991; Skene, 1991; Winick, 1991; Schopp, 1994; Wilks, 1997, 1999; Grisso and Appelbaum, 1998; Saks, 1999; Berghmans, 2001; Buchanan, 2004; Howe, 2010; Kim, 2010; Bolt and van Summeren, 2014; den Hartogh, 2016; Roberts, 2018
	*Epistemic* The amount of evidence or confidence required for a finding of competence should vary with risk	12	Wicclair, 1991a,b, 1999; Cale, 1999; Checkland, 2001; DeMarco, 2002; Parker, 2004, 2006; Brudney and Siegler, 2015; Manson, 2015; Lawlor, 2016; Graber, 2021
	*Neither Substantive nor Epistemic* Does not endorse either view	7	Kloezen et al., 1988; Culver and Gert, 1990; Elliott, 1991; Saks, 1991; White, 1994; Maclean, 2000; Buller, 2001

Some publications endorsed both the substantive and epistemic views. These publications were categorized under substantive view, as most of the debate about RS-DMC focuses on whether the substantive view of RS-DMC is appropriate. For instance, articles critical of RS-DMC always targeted the substantive view, never the epistemic. In many cases, articles critical of substantive RS-DMC endorsed the epistemic view. More specifically, the epistemic view is often used to allow for the inclusion of risk in DMC assessment, thus satisfying the common intuition that this is appropriate, while avoiding the supposed flaws of the substantive view. Thus, the debate largely is between those who support the substantive view and those who do not, regardless of whether they support the epistemic view. Accordingly, hereafter we will use ‘RS-DMC' to refer to the substantive view of RS-DMC, unless otherwise specified.

Further, there is debate in the literature about the distinction between substantive and epistemic RS-DMC. Some authors question whether these views differ in practice (Wilks, 1999), while others maintain that there is a significant distinction (Wicclair, 1999; Parker, 2004).

### Reasons Used in the Debate

We identified 26 primary reasons in the literature that were used in more than one publication (Tables 2–4). We classified 8 reasons as “Pros” (Table 2), 10 reasons as “Cons” (Table 3), and 8 reasons as “Counterarguments Defending RS-DMC” (Table 4). This reflects the progression of the debate over time.

**Table 2:** Pro arguments.

**Code**	**Argument type**	**Argument content and examples**	***N***	**References**
P1	Coheres with current practice	RS-DMC coheres with current medical and/or legal practice and norms or common understanding in every day sense of competence “a concept that allows a raising or lowering of the standard for decision-making capacities depending upon the risks of the decision in question is clearly more consonant with the way people actually make informal competency determinations” (Buchanan and Brock, 1989)	17	Drane, 1985; Buchanan and Brock, 1986, 1989; Feinberg, 1986; Brock, 1991; Skene, 1991; Winick, 1991; Schopp, 1994; Wilks, 1997, 1999; Grisso and Appelbaum, 1998; Buchanan, 2004; Howe, 2010; Kim, 2010; Lawlor, 2016; Graber, 2021
P2	Balances autonomy and welfare	RS-DMC is the best way to balance the competing values of autonomy/self-determination and well-being/welfare “It allows a better balance between the competing values of self-determination and well-being that are to be served by a determination of competence” (Buchanan and Brock, 1986)	14	Drane, 1984, 1985; Buchanan and Brock, 1986, 1989; Eastman and Hope, 1988; Brock, 1991; Winick, 1991; Grisso and Appelbaum, 1998; Berghmans, 2001; Kim, 2010; Bolt and van Summeren, 2014; Brudney and Siegler, 2015; den Hartogh, 2016; Lawlor, 2016
P3	Balances potential errors	RS-DMC balances two potential errors: (1) authorizing an incompetent patient's decision, leading to harm and (2) overruling a competent patient's decision, disrespecting their autonomy. As risk increases, (1) is more damaging than (2), requiring that the standard for deeming a patient competent increases. “A properly performed competency assessment should eliminate two types of error: (1) preventing a competent person from participating in treatment decisions and (2) failing to protect an incompetent person from the harmful effects of a bad decision.” (Drane, 1984)	7	Drane, 1984, 1985; Buchanan and Brock, 1986, 1989; Schopp, 1994; Berghmans, 2001; Buchanan, 2004
P4	Rejecting fixed level of competence safeguards against broader paternalism	If there is one fixed level of competence that applies to all situations, it has broader paternalistic consequences. “the alternative would be to let go of the presumption of competence itself, and examine patients' competence in all cases, whether or not there is any reason for doubt. That would really be paternalistic in the extreme” (den Hartogh, 2016).	5	Drane, 1984; Buchanan and Brock, 1986, 1989; Winick, 1991; den Hartogh, 2016
P5	Avoids unnecessarily burdening the system	If every impaired person is interrogated or held to some high standard relative to the risk, then system would be burdened with very little gained. “it … would expend scarce resources without achieving significant benefits” (Winick, 1991)	4	Drane, 1984, 1985; Winick, 1991; Brudney and Siegler, 2015
P6	Respecting patients' wishes has value	RS-DMC allows for lowering the standard for DMC and respecting patient's wishes in low-risk situations, which is valuable “applying a lower standard of competency when a patient assents to a recommended course of treatment than when a patient objects, serves not only individual autonomy values, but also the interest in promoting health.” (Winick, 1991)	2	Winick, 1991; Buchanan, 2004
P7	The opposing view needs to articulate a natural ‘adequate level' of decision-making abilities	If risk is not used in setting a threshold for DMC, a fixed standard must be identified and defended, but no such model exists. “On both views a certain point on the scale of competence can be identified at which we are prepared to attribute that authority in a particular case, even if, on the multi-dimensional view, we also have to take other considerations into account in order to do that. How do we identify that point? I will argue that until now only the multi-dimensional theory has been able to provide a plausible answer to that question.” (den Hartogh, 2016)	2	Brock, 1991; den Hartogh, 2016
P8	Tailored DMC assessment	RS-DMC allows for DMC assessment to be tailored to the needs of each patient “The use of a sliding scale allows care providers to tailor the standard they use to the particular needs of each patient” (Howe, 2010)	2	Howe, 2010; Bolt and van Summeren, 2014

### Reasons in Favor of RS-DMC

The most common reasons in favor of RS-DMC rely on the intuitive appeal of RS-DMC and its practical merits (Table 2). For example, many publications argue that RS-DMC allows for a balance between autonomy and welfare (P2) and the balancing of potential errors (P3), but generally do not explain this balancing in detail, although there are exceptions (Schopp, 1994; Buchanan, 2004). Similarly, “Coheres with Current Practice” (P1), “Rejecting fixed level of competence safeguards against broader paternalism” (P4), “Avoids unnecessarily burdening the system” (P5), and “The opposing view needs to articulate a natural ‘adequate level' of decision-making abilities” (P7) focus on the practical difficulties that would result from rejecting RS-DMC. Finally, though cited infrequently, “Respecting patient's wishes has value” (P6) and “Tailored DMC assessment” (P8) do not fall neatly into either intuitive appeal or practical merits.

### Reasons Against RS-DMC

Most of the reasons against RS-DMC reflect concerns about paternalism (Table 3, arguments C2 through C9). Some arguments are direct charges of paternalism (“RS-DMC is paternalistic” [C2]) while others imply this concern. For example, “Form of outcome-based DMC” (C3) is the argument that when risk is incorporated into DMC assessment, a patient may be deemed incompetent and have their decisions ignored based on merely the decision they make. Similarly, “Imports assessor's values” (C6), “Introduction of values into value-neutral assessment” (C8), and “Falsely finds incompetent persons competent” (C9) are rooted in concerns that DMC assessment will be driven by physicians' values rather than any objective measure, and patients will be judged on whether their decisions/values differ from those of the evaluating physician. Finally, “Conflation of DMC and Decisional Authority” (C4) and “RS-DMC is tautological” (C5) argue that RS-DMC is conceptually flawed and only nominally protects patient autonomy, as a physician could seemingly deem a patient “incompetent” as long as he deemed the risk high enough, rather than assess competence with no consideration of risk and independently decide whether the patient's decision should be respected.

**Table 3:** Con arguments.

**Code**	**Argument type**	**Argument content and examples**	***N***	**Authors represented**
C1	Asymmetry between consent and refusal	Asymmetry between consent and refusal is conceptually incoherent or problematic “Extant accounts of risk- related standards of capacity appear to be committed to the existence of asymmetrical capacity, that is, cases where a patient is capacitated to accept treatment but lacks capacity to reject treatment. However, asymmetrical capacity appears to be conceptually incoherent; in such cases, there is no sense to be made of the claim that the patient either has, or lacks, capacity.” (Graber, 2021)	11	Culver and Gert, 1990; Wicclair, 1991a,b, 1999; Cale, 1999; Maclean, 2000; Berghmans, 2001; Buller, 2001; Manson, 2015; Lawlor, 2016; Graber, 2021
C2	RS-DMC is paternalistic	RS-DMC is inherently paternalistic and inconsistent with autonomy, or is highly prone to paternalistic abuse by allowing evaluator to set threshold according to their own values “[T]here is a danger that standards of understanding, reasoning, and so forth will be set arbitrarily and unattainably high by those who believe that paternalism is justified when perceived risks are great.” (Wicclair, 1991a)	11	Culver and Gert, 1990; Saks, 1991; Wicclair, 1991a,b; White, 1994; Cale, 1999; Maclean, 2000; Berghmans, 2001; DeMarco, 2002; Buchanan, 2004; Parker, 2004
C3	Form of outcome-based DMC	DMC assessment should be process-oriented and should not depend on the likely outcome of the choice an individual makes. “their account appears to be incompatible with the principle that assessments of decision-making capacity should utilize a standard that is process-oriented, and not result-oriented.” (Wicclair, 1991b)	9	Wicclair, 1991a,b; White, 1994; Cale, 1999; Saks, 1999; Maclean, 2000; Buller, 2001; Parker, 2004; Saks and Jeste, 2006
C4	Conflation of DMC and DA	RS-DMC conflates two distinct judgments: (1) whether a person has DMC/competence and (2) whether their decision should have authority “The sliding-scale model of competence based on risk conflates two different questions: (1) whether the patient is competent, and (2) whether we should respect the patient's decision” (Elliott, 1991)	8	Culver and Gert, 1990; Elliott, 1991; Wicclair, 1991a, 1999; White, 1994; Berghmans, 2001; Buller, 2001; DeMarco, 2002
C5	Respecting competent patient's decision is a tautology	If a variable standard is used, prohibition of paternalism (overriding a competent patient's decision) is a mere tautology rather than a strong commitment to patient autonomy “Since the statement that the treatment preferences of competent patients are not to be set aside for paternalistic reasons amounts to a tautology, it hardly reflects a strong commitment to the ethical principle that treatment choices of autonomous patients should be respected.” (Wicclair, 1991a)	8	Culver and Gert, 1990; Elliott, 1991; Wicclair, 1991a,b, 1999; Maclean, 2000; DeMarco, 2002; den Hartogh, 2016
C6	Imports assessor's values	RS-DMC relies on the assessor's judgment and values over those of the patient “[T]his manner of assessing competency allows the evaluator to determine that a choice is problematic based upon his or her own values” (Saks, 1999)	8	Saks, 1991, 1999; White, 1994; Cale, 1999; Maclean, 2000; Parker, 2004; Saks and Jeste, 2006; Manson, 2015
C7	Standards vary with complexity, not risk	Complexity of decisions, not risk, explains our intuitions about high-risk decision-making “There may be a correlation between greater risk and increased complexity of requisite decision making skills and abilities” (Wicclair, 1999)	5	Kloezen et al., 1988; Wicclair, 1991a, 1999; Maclean, 2000; Berghmans, 2001
C8	Introduction of values into value-neutral assessment	DMC assessment should be value neutral, but RS-DMC introduces normative values into assessment “understanding competence as related to outcomes requires the unjustified imposition of normative values in the assessment of competence, thereby confusing the kind of competence that a standard is aimed at assessing” (Cale, 1999)	4	White, 1994; Cale, 1999; DeMarco, 2002; Parker, 2004
C9	RS-DMC falsely finds incompetent persons competent	RS-DMC allows those who lack the abilities required to make decisions to be deemed competent or accountable in low-risk situations “As a result, there is the danger that decision-making standards will be set so low when patients concur with the recommendations of health care professionals that they will be classified as decisionally capable, regardless of their mental status.” (Wicclair, 1991a)	4	Wicclair, 1991a,b; Berghmans, 2001; Lawlor, 2016
C10	Coherence is not sufficient reason	Legal or medical coherence is not a good reason, or the status quo is problematic “At any rate, it seems a poor reason to adopt a misleading definition of a concept to say it accords better with a legal tradition that is itself vague and confused.” (Culver and Gert, 1990)	3	Culver and Gert, 1990; Saks, 1991; DeMarco, 2002
C11	It is unclear where to set the threshold	When risk is included, it is unclear where the threshold for DMC should be set “First, let us note that the question of what different standards of capacity would actually look like never arises in most of the risk-related accounts. All we hear is that in cases of higher risk, a higher standard of decision-making capacity is required.” (Parker, 2004)	2	Kloezen et al., 1988; Parker, 2004

“Standards vary with complexity, not risk” (C7), “Coherence is not sufficient reason” (C10), and “It is unclear where to set the threshold” (C11) do not fit as neatly into this umbrella of concern over paternalism. C10 denies that coherence with current practice is sufficient reason for RS-DMC. C11 highlights a practical concern about RS-DMC implementation. C7 is a disagreement over what explains the intuitive appeal of raising the threshold when decisions are high-risk, but it is disputed by even some critics of RS-DMC.

The asymmetry between consent and refusal (C1) is the most discussed argument regarding RS-DMC. It says that RS-DMC implies the conceptually incoherent view that a person can be competent to consent but not to refuse. Some argue that even if this is not conceptually incoherent, it leads to ethically unacceptable evaluations involving bad faith or deception (Lawlor, 2016).

### Counterarguments Against Criticisms of RS-DMC

Most of the counterarguments defend RS-DMC against charges of paternalism (Table 4). Some do so by directly refuting the criticisms (“RS-DMC is not paternalistic,” [CA2], “RS-DMC is not tautological,” [CA5]), but others defend RS-DMC by clarifying how the model functions in practice or by disputing the conceptual premises of the critics. For example, “Outcome alone does not determine DMC” (CA3) clarifies that RS-DMC may include risk or outcome as *part* of the assessment, but does not rely on these factors alone; therefore, outcome alone does not determine DMC as is suggested by critics. Similarly, “Consistent with the reasonable person standard” (CA6) points out how consideration of risk is consistent with the commonly used reasonable person standard, and thus does not import values inappropriately. “Not a conflation” (CA4) argues that the disagreement is due to differences in the conceptual frameworks of decision-making capacity used. Similarly, “Inherently normative” (CA8) asserts the position that DMC assessment cannot be value-neutral.

**Table 4:** Counterarguments.

**Code**	**Argument type**	**Argument content and examples**	***N***	**Authors represented**
CA1*	Asymmetry is not problematic or needed	(a) Asymmetry is admittedly odd but cost is acceptable; “There is an important implication of this view that the standard of competence ought to vary with the expected harms or benefits to the patient of acting in accordance with a choice–namely, that just because a patient is competent to consent to a treatment, it does not follow that the patient is competent to refuse it, and vice versa.” (Buchanan and Brock, 1986) (b) Consenting and refusing are separate decisions, so there is no asymmetry in RS-DMC “One reason a patient might be competent to consent but not to refuse a treatment, and vice versa, is that the two choices to consent or refuse will be based on different processes of reasoning or decision-making; the overall processes of reasoning must be different if for no other reason than that they result in different choices.” (Brock, 1991) (c) Asymmetry is about presumption of capacity, and is actually justified “the greater the risk to the patient, the more reason the physician has to think about capacity.” (Brudney and Siegler, 2015) (d) If (c) is accepted, then asymmetry is not needed for RS-DMC “It may be that if the patient consents there is no reason to investigate his competence, but if he refuses, there is. However, if the conclusion following from that investigation is negative, it holds for the consent as well as for the refusal.” (den Hartogh, 2016)	15	Buchanan and Brock, 1986, 1989; Brock, 1991; Winick, 1991; Wilks, 1997, 1999; Berghmans, 2001; Checkland, 2001; Howe, 2010; Kim, 2010; Bolt and van Summeren, 2014; Brudney and Siegler, 2015; den Hartogh, 2016; Lawlor, 2016; Graber, 2021
CA2	RS-DMC is not paternalistic	Any argument that claims RS-DMC is not paternalistic “it will generally be the case that if the patient's decision does not coincide with the opinion of the physician, this may trigger the need to assess the patient's capacity. This, however, should not be confused with ‘lowering the bar' for incapacity.” (Berghmans, 2001)	13	Drane, 1984, 1985; Buchanan and Brock, 1986, 1989; Feinberg, 1986; Eastman and Hope, 1988; Winick, 1991; Schopp, 1994; Wilks, 1997; Berghmans, 2001; Kim, 2010; Roberts, 2018; Graber, 2021
CA3	Outcome alone does not determine DMC	RS-DMC may include the outcome/choice itself as indicative of risk, but other factors are also essential to DMC assessment. “But outcome is not the standard of competence in this model. Rather it is an important factor in only one class of medical decisions.” (Drane, 1985)	10	Drane, 1985; Buchanan and Brock, 1986, 1989; Eastman and Hope, 1988; Winick, 1991; Schopp, 1994; Wilks, 1997; Saks, 1999; Berghmans, 2001; Buchanan, 2004
CA4	Not a conflation but different framework of DA	RS-DMC is a conflation of DMC and DA only if you believe DMC is purely a matter of abilities; if you accept that the function of DMC assessment is to determine DA, it is not a conflation “Wicclair insists our account conflates two distinct questions - first, is the patient competent to make the decision and, second, is there reason to disregard the patient's decision and have a surrogate decide for the patient. As we discussed (65–70), an alternative account of competence is possible in which these two questions are distinguished. Competence would then be understood as requiring some minimum threshold of decisionmaking capacities, though the threshold could still be decision-specific and variable, but not as determining decisional authority. This account would leave open whether a patient's competent choice should be set aside on paternalistic grounds in order to protect his or her well-being. We called this a two-step model of patient decision-making authority. We evaluated such a model and argued that our own account was preferable” (Brock, 1991)	5	Buchanan and Brock, 1989; Brock, 1991; Skene, 1991; Berghmans, 2001; Bolt and van Summeren, 2014
CA5	RS-DMC is not tautological	Any argument that responds to the criticism that RS-DMC makes respecting a competent patient's decision tautological “Is our view problematic and empty of any commitment to individual self-determination in this way? It would be if we offered no other criteria for a justified finding of incompetence than that others believed setting aside patients' treatment choices for their own good was justified.” (Brock, 1991)	2	Brock, 1991; Wilks, 1997
CA6	RS-DMC is consistent with the reasonable person standard	Risk consideration does not import assessor's values, as it is consistent with the commonly accepted ‘reasonable person standard'. “[T]reatment refusal does reasonably raise the question of a patient's competence in a way that acceptance of recommended treatment does not. It is a reasonable assumption that physicians' treatment recommendations are more often than not in the interests of their patients. Consequently, it is a reasonable presumption-though rebuttable in any particular instance- that a treatment refusal is contrary to the patient's interest.” (Buchanan and Brock, 1986)	10	Drane, 1985; Buchanan and Brock, 1986, 1989; Feinberg, 1986; Skene, 1991; Winick, 1991; Brudney and Siegler, 2015; Lawlor, 2016; Graber, 2021
CA7	Complexity alone can't explain variable standard	Riskier decisions are not necessarily more complex, so risk itself must be what is responsible for the intuitive appeal of variable thresholds “However, complexity is not the same thing as risk: a high-risk procedure may be extremely straightforward, and a low-risk procedure could be quite complicated.” (Parker, 2004)	7	Brock, 1991; Skene, 1991; White, 1994; Wilks, 1997; Buller, 2001; Parker, 2004; Kim, 2010
CA8^#^	DMC assessment is inherently normative	It is impossible for DMC assessment to be value-neutral; it naturally relies on normative judgments “Given the uncertainty and inherent vagueness of the criteria to be applied, physicians assigned the task of assessing competency inevitably make normative judgments.” (Winick, 1991)	10	Winick, 1991; Wilks, 1997, 1999; Grisso and Appelbaum, 1998; Saks, 1999; Berghmans, 2001; Saks and Jeste, 2006; Kim, 2010; Bolt and van Summeren, 2014; den Hartogh, 2016

*Counterarguments are numbered the same as the con argument to which they respond.

*^#^We did not find any counterarguments directly responding to C9, C10, or C11*.

The counterarguments against the asymmetry argument (CA1), however, are quite varied. Some simply acknowledge that the asymmetry seems odd but embrace it as part of RS-DMC (Buchanan and Brock, 1986; Howe, 2010), or consider consenting and refusing two separate decisions^3^ (Brock, 1991; Wilks, 1997). Others show that asymmetry appears acceptable when determining whether to maintain or rebut the presumption of capacity in a given situation (Checkland, 2001; Brudney and Siegler, 2015). Other supporters of RS-DMC argue further that aside from the context of evaluating the presumption of capacity, RS-DMC not only does not need but should not include asymmetry of consent and refusal (Bolt and van Summeren, 2014; den Hartogh, 2016; Graber, 2021).

### Underlying Frameworks Used by Authors

Authors varied on how competence and related concepts are defined or understood. We use “frameworks” to refer to structural or content features of the author's conception of competence and related concepts (Table 5). We categorized conservatively: a publication was a given category only if it explicitly endorsed or clearly made use of a particular framework or definition; thus, it is possible an author in fact holds a certain framework but we could not code a publication as such.

**Table 5:** Frameworks used.

**Framework**	**Definition**	***N***	**References**
Externalist or Internalist	*Externalist* Competence judgment determined by both internal abilities and other contextual or relational factors external to the abilities.	17	Buchanan and Brock, 1986, 1989; Feinberg, 1986; Eastman and Hope, 1988; Brock, 1991; Winick, 1991; Wilks, 1997, 1999; Grisso and Appelbaum, 1998; Berghmans, 2001; Buchanan, 2004; Saks and Jeste, 2006; Kim, 2010; Bolt and van Summeren, 2014; den Hartogh, 2016; Lawlor, 2016; Roberts, 2018
	*Internalist* Competence judgment determined solely by level of abilities within the person	9	Kloezen et al., 1988; Culver and Gert, 1990; Wicclair, 1991a,b, 1999; White, 1994; Cale, 1999; Maclean, 2000; Checkland, 2001
One step or two step	*One step* Arriving at DA judgment is a single step that incorporates information about P's abilities plus contextual factors (e.g., risk).	12	Buchanan and Brock, 1986, 1989; Feinberg, 1986; Eastman and Hope, 1988; Brock, 1991; Skene, 1991; Winick, 1991; Grisso and Appelbaum, 1998; Buchanan, 2004; Kim, 2010; Bolt and van Summeren, 2014; den Hartogh, 2016
	*Two step* Evaluators should assess an individual's abilities in order to reach a competence judgment; then, decide whether the person has decisional authority	7	Culver and Gert, 1990; Elliott, 1991; Wicclair, 1991a,b, 1999; Maclean, 2000; Buller, 2001
Does having DMC imply having DA?	*Yes* A finding of DMC gives an individual DA, so their decisions should be respected	17	Buchanan and Brock, 1986, 1989; Feinberg, 1986; Eastman and Hope, 1988; Kloezen et al., 1988; Brock, 1991; Skene, 1991; Winick, 1991; White, 1994; Wilks, 1997; Grisso and Appelbaum, 1998; Checkland, 2001; Buchanan, 2004; Kim, 2010; Bolt and van Summeren, 2014; den Hartogh, 2016; Roberts, 2018
	*No* A finding of DMC means the person has the ability to make decisions. Whether their decision should be respected is a separate judgment.	7	Elliott, 1991; Wicclair, 1991a,b, 1999; Maclean, 2000; Buller, 2001
Conception of well-being	*Objective* Author emphasizes or uses objective or shared meaning of welfare.	15	Drane, 1984, 1985; Eastman and Hope, 1988; Culver and Gert, 1990; Winick, 1991; Schopp, 1994; Wilks, 1997; Grisso and Appelbaum, 1998; Buchanan, 2004; Saks and Jeste, 2006; Kim, 2010; Bolt and van Summeren, 2014; Manson, 2015; den Hartogh, 2016; Lawlor, 2016
	*Subjective* Author emphasizes or uses individual subject's own meaning of welfare or value.	6	Buchanan and Brock, 1986; White, 1994; Grisso and Appelbaum, 1998; Cale, 1999; Buller, 2001; Roberts, 2018; Graber, 2021
	*Both*	9	Feinberg, 1986; Skene, 1991; Saks, 1999; Wilks, 1999; Maclean, 2000; DeMarco, 2002; Howe, 2010
Specific decision or type of decision	*Specific decision* The specific decision a person makes is relevant to competence assessment	17	Drane, 1984, 1985; Buchanan and Brock, 1986; Feinberg, 1986; Eastman and Hope, 1988; Brock, 1991; Winick, 1991; Grisso and Appelbaum, 1998; Wicclair, 1999; Berghmans, 2001; Saks and Jeste, 2006; Howe, 2010; Kim, 2010; Bolt and van Summeren, 2014; den Hartogh, 2016; Lawlor, 2016; Graber, 2021
	*Type of decision* Competence assessment is about the person's more general decision-making abilities, not the specific decision they make.	7	Kloezen et al., 1988; Culver and Gert, 1990; Saks, 1991, 1999; Cale, 1999; Maclean, 2000; Buller, 2001

The first framework category of “Externalism” vs. “Internalism” is a distinction noted by some authors (Wilks, 1997; Berghmans, 2001). Internalism holds that competence is solely a function of a person's internal abilities relevant to decision-making; externalism holds that competence is determined by both internal abilities and other contextual or relational factors external to the person's decisional abilities, such as risk. Of those publications codable on this issue, most were externalist (17/26).

A closely related framework category—“One-step” vs. “Two-step”—addresses how decisional authority should be determined (Buchanan and Brock, 1986; Culver and Gert, 1990). The two options are either a single step that incorporates information about a patient's abilities in addition to relevant contextual factors such as risk or taking two steps, first determining competence, then separately determining whether the person should have decisional authority. Of the 19 publications codable on this issue, most held the one-step view (12/19).

Similarly, the third framework category asks, “Does having DMC imply having decisional authority?” Some hold that a finding of DMC grants an individual decisional authority, so their decisions must be respected, while others hold that a finding of DMC only means that an individual has the abilities to make decisions, not that they should automatically have decisional authority. The majority (17/24) endorsed the view that having DMC implies having decisional authority. The first three framework categories seem closely related conceptually.

The fourth framework category is the *predominant* conception of well-being that a publication relies on in its model of DMC. There were three coding options: (1) predominantly objective, when publications primarily use an objective or shared understanding of welfare, (2) predominantly subjective, when publications primarily use an individual's own subjective understanding of their welfare, and (3) both objective and subjective used, with neither clearly favored over the other. Among codable documents on this issue, half (15/30) had a predominantly objective conception, while 9/30 relied on both, and the remaining 6/30 relied primarily on a subjective perspective.

Finally, publications were categorized according to their view on the scope of competence, that is, whether competence is about a *specific, particular* decision or about a *type* of decision. Those publications that hold the first view argue that the specific decision a person makes is relevant to competence assessment, while those that hold the second view argue that an individuals' competence should instead depend on an individual's ability to make the relevant *type* of decision. 17/24 codable publications on this issue supported the “specific decision” view.

### Relationship of Frameworks to Stance on RS-DMC

The framework categories identified in Table 5 are highly associated with an author's stance on RS-DMC.

For example, 16 of 17 publications that hold an externalist view endorse the substantive view of RS-DMC, while all 9 publications that hold an internalist view do not (Table 6). Similarly, all 12 publications that support a one-step determination of decisional authority endorse the substantive view, 12 out of 15 publications that use an objective conception of well-being support the substantive view, and 14 out of 17 publications that use “competence of specific decision” support the substantive view.

**Table 6:** Relationship of frameworks to stance on RS-DMC.

**Framework**	**View**	**Substantive % (*N*)**	**Epistemic only % (*N*)**	**Neither S nor E % (*N*)**
Externalism vs. Internalism	Externalist view of DMC	94.1 (16/17)	5.9 (1/17)	0 (0/17)
	Internalist view of DMC	0 (0/9)	55.6 (5/9)	44.4 (4/9)
	Uncoded	40 (6/15)	40 (6/15)	20 (3/15)
One-step vs. Two-step	One-step determination of DA	100 (12/12)	0 (0/12)	0 (0/12)
	Two-step determination of DA	0 (0/7)	42.9 (3/7)	57.1 (4/7)
	Uncoded	45.5 (10/22)	40.9 (9/22)	13.6 (3/22)
Does having DMC imply having	Having DMC implies having decisional authority	82.4 (14/17)	5.9 (1/17)	11.8 (2/17)
decisional authority?	Having DMC does not imply having decisional authority	0 (0/7)	42.9 (3/7)	57.1 (4/7)
	Uncoded	47.1 (8/17)	47.1 (8/17)	5.9 (1/17)
Conception of wellbeing	Objective wellbeing	80 (12/15)	13.3 (2/15)	6.7 (1/15)
	Subjective wellbeing	33.3 (2/6)	33.3 (2/6)	33.3 (2/6)
	Both	66.7 (6/9)	22.2 (2/9)	11.1 (1/9)
	Uncoded	18.2 (2/11)	54.5 (6/11)	27.3 (3/11)
Specific decision or type of decision	Competence of specific decision	82.4 (14/17)	17.6 (3/17)	0 (0/17)
	Competence of type of decision	14.3 (1/7)	14.3 (1/7)	71.4 (5/7)
	Uncoded	41.2 (7/17)	47.1 (8/17)	11.8 (2/17)

### Relationship of Frameworks to Reasons

The frameworks are sometimes also associated with reasons for or against RS-DMC. This was most obvious when there was a logical connection between the frameworks and the reasons. For example, the two closely inter-related framework elements of “two step vs. one step” view of decisional authority and whether DMC implies having decisional authority were highly associated with two arguments against RS-DMC, namely, whether an author criticized RS-DMC as conflating DMC with decisional authority (C4) and as providing only tautological prohibition of paternalism (C5). For example, 5/7 publications that hold that having DMC does not imply having decisional authority argue that RS-DMC involves a conflation of DMC and decisional authority (C4), whereas authors who view DMC as implying decisional authority understandably do not see a conflation (0 among 12 codable papers). Similarly, 6/7 publications that hold a two-step view of DMC assessment and 6/7 publications that hold that having DMC does not imply having decisional authority argue that RS-DMC prohibits paternalism by definition only (“RS-DMC is tautological” [C5]). These findings suggest that at least some areas of debate over RS-DMC arise due to differing underlying premises.

## Discussion

The concept of DMC is widely used every day in most jurisdictions, yet it still engenders debate and disagreement. One particularly controversial debate is whether DMC assessment should be risk-sensitive. This debate began in earnest in 1984, yet remains controversial. Our review of the arguments used in the debate reveal several key findings.

### The Importance of Frameworks

There is a lack of uniformity in vocabulary and definitions in the debate. The publications were in journals from a variety of disciplines, by authors with diverse backgrounds and across jurisdictions, which may explain some of the differences in vocabulary. But authors often understand the concepts of DMC and decisional authority differently, as captured by the five framework categories that we tracked in the literature (Table 5).

These differences can make it unclear whether disagreement reflects misunderstanding or substantive ethical disagreement. It appears at least some of the disagreements about RS-DMC may be due to differences in frameworks and premises^4^. The frameworks endorsed also sometimes relate to the reasons each publication used. For example, “Conflation of DMC and decisional authority” (C4) or the tautology argument (C5) require the view that having DMC does not imply having decisional authority, a minority view in the literature. The significance of frameworks has been relatively neglected in the debate. Future debates on RS DMC may benefit from explicit attention to this issue.

### Patterns in Reasons Used

Though there are many distinct reasons for and against RS-DMC in the literature, a few broad patterns became apparent. First, the pro RS-DMC arguments tend to rely on the intuitive and practical appeal of RS-DMC. Second, arguments against RS-DMC mostly have to do with two concerns: one, concerns about paternalism (although sometimes this is only implicit) and, two, concern about the coherence of asymmetry of consent and refusal that is said to be part of RS-DMC. Finally, the most notable feature of the counterarguments defending RS-DMC (aside from defending against the variety of charges of paternalism) is that there were a variety of responses to the asymmetry argument with differing views among defenders of RS-DMC. Given that the RS-DMC debate has perhaps focused more on the asymmetry argument than any other issue, this is an interesting finding and suggests that further research is needed.

### Future Work

In addition to the issue of asymmetry, future work should focus more on the distinction between the substantive and epistemic views of RS-DMC. It is curious that those critical of RS-DMC often permit the incorporation of risk into DMC assessment through the epistemic view, rather than arguing risk should be entirely irrelevant. Thus, the intuitive appeal of risk-sensitivity carries a significant weight even among interlocutors who disagree with the substantive view of RS-DMC.

Additionally, it is notable that no publications critical of risk-sensitive DMC assessment targeted the epistemic view. However, whether there is a practical difference between the epistemic vs. substantive view of RS-DMC is disputed (Wicclair, 1999; Wilks, 1999; Parker, 2004). Given how differently each view is treated in the debate, clarification of the precise differences between the views would be valuable.

### Limitations

Both construction and application of codes require judgment and interpretation. For example, some codes have significant conceptual overlap—e.g., “Does having DMC imply having decisional authority” and “Two-step vs. One-step”—but we felt it was important to track both codes separately to capture their nuances, particularly because, in order to minimize bias, we coded conservatively and only coded a reason when it was explicit. Our inclusion of books in addition to our systematic search of articles is a further limitation, as a systematic search of books was not possible. However, it seems unlikely that this led to our missing any major arguments or reasons for or against RS-DMC. Only publications written in English were included in our search, and most of the publications included are from the US or UK. Literature in other languages or published in other countries may provide different perspectives on RS-DMC. For example, the emphasis on autonomy may vary among different cultures (Lepping and Raveesh, 2014), and this could also affect views regarding RS-DMC.

## Conclusion

Whether assessment of DMC should be risk-sensitive is an important and hotly contested issue. We find that some of the debate stems from differences in underlying conceptual frameworks of the authors, as the frameworks are highly associated with one's stance on RS-DMC. Most positive defenses of RS-DMC rely on its intuitive appeal, while most criticisms are driven by concern about paternalism or the asymmetry between consent and refusal. It is notable that defenders of RS-DMC address the asymmetry concern in a variety of ways, suggesting that more attention to this issue is needed. Future work should also clarify the differences between the epistemic and substantive views of RS-DMC.

## Data Availability Statement

The original contributions presented in the study are included in the article/Supplementary Material, further inquiries can be directed to the corresponding author/s.

## Author Contributions

NB and SK both contributed to project conception and design, screening and coding of manuscripts, and preparing and editing manuscript drafts. All authors contributed to the article and approved the submitted version.

## Funding

This research was supported in part by the Intramural Research Program of the National Institutes of Health Clinical Center (CL010542).

## Author Disclaimer

The ideas and opinions expressed are the authors'; they do not represent any position or policy of the National Institute of Health, the Department of Health and Human Services, or the U.S. government.

## Conflict of Interest

The authors declare that the research was conducted in the absence of any commercial or financial relationships that could be construed as a potential conflictof interest.

## Publisher's Note

All claims expressed in this article are solely those of the authors and do not necessarily represent those of their affiliated organizations, or those of the publisher, the editors and the reviewers. Any product that may be evaluated in this article, or claim that may be made by its manufacturer, is not guaranteed or endorsed by the publisher.

## Footnotes

^1^Although the most common context for DMC evaluation is medical, we did not limit our search to this context.

^2^Note that a publication that clearly argues for RS-DMC could still include individual arguments against RS-DMC. Thus, individual arguments attributed to a publication do not necessarily reflect a publication's overall stance on RS-DMC.

^3^The question of whether or not consenting and refusing should be considered two separate decisions appears in the law as well. For example, the Mental Capacity Act of 2005 in England and Wales states that “the courts do not examine separately capacity to consent and capacity to refuse medical treatment. Rather, the courts proceed by examining the question of whether the person has the capacity to make a decision in relation to the treatment.

^4^Does one's view on RS-DMC determine one's framework elements or vice versa? This is not an easy question to answer. For example, although it is true that RS-DMC logically implies an externalist view of DMC and therefore one could argue that externalism/internalism element is an implication rather than a premise of RS-DMC, it is not so obvious whether RS-DMC requires a specific view about whether DMC implies decisional authority.
