# Supplementary material for: Should Assessments of Decision-Making Capacity Be Risk-Sensitive? A Systematic Review
Source: Front Psychol. 2022 Jun 29;13:897144. doi: 10.3389/fpsyg.2022.897144 (PMC9277305; doi:10.3389/fpsyg.2022.897144)
Supplement: Supplementary file 1 [file Data_Sheet_1.DOCX]

**Appendix A**

**PubMed/MEDLINE (US National Library of Medicine) Exact Search**

("sliding scale*"[tiab] OR "risk sensitive"[tiab] OR "risk sensitivity"[tiab] OR "risk-related"[tiab] OR “Risk relativity”[tiab] OR "variable standard*"[tiab] OR "variable threshold*"[tiab] OR paternalism[tiab] OR paternalistic[tiab] OR paternalism[Mesh])  AND  (competence[tiab] OR competency[tiab] OR "mental capacity"[tiab] OR "mental capacities"[tiab] OR "decision-making capacit*"[tiab] OR incompetenc*[tiab] OR “decisional authorit*"[tiab] OR “Diminished capacit*”[tiab] OR "Mental Competency"[Mesh]) AND english[lang]

485 results on 02/15/22

**PsycInfo (American Psychological Association) Exact Search**

**The PsycInfo search was constructed as follows:**

1. ("sliding scale" OR “sliding scales” OR "risk sensitive" OR "risk sensitivity" OR "risk-related" OR “Risk relativity” OR "variable standard" OR “variable standards” OR "variable threshold" OR “variable thresholds” OR paternalism OR paternalistic) in Title
2. ("sliding scale" OR “sliding scales” OR "risk sensitive" OR "risk sensitivity" OR "risk-related" OR “Risk relativity” OR "variable standard" OR “variable standards” OR "variable threshold" OR “variable thresholds” OR paternalism OR paternalistic) in Abstract
3. ({Paternalism} OR {Minimum Competency Tests}) in Index Terms
4. 1) or 2) or 3)
5. (competence OR competency OR "mental capacity" OR "mental capacities" OR "decision-making capacity" OR “decision-making capacities” OR incompetence OR incompetency OR “decisional authority" OR “decisional authorities” OR “Diminished capacity” OR “diminished capacities”) in Title
6. (competence OR competency OR "mental capacity" OR "mental capacities" OR "decision-making capacity" OR “decision-making capacities” OR incompetence OR incompetency OR “decisional authority" OR “decisional authorities” OR “Diminished capacity” OR “diminished capacities”) in Abstract
7. ({Diminished Capacity} OR {Competence} OR {Decision Making}) in Index Terms
8. “Mental Competency” in MeSH terms
9. 5) OR 6) OR 7) OR 8)
10. 4) AND 9)
11. 10) AND English Language
12. 11) AND Peer Reviewed Journals Only

495 results on 02/15/2022

**PhilPapers Exact Search**

(“risk” | "risk-sensitive" | "risk-related" | "sliding-scale" | "variable standard"| "paternalism" ) & (competence | capacity | "decision-making capacity")

111 results 02/15/2022
